# Methodological Reporting Quality of Randomized Controlled Trials in 3 Leading Diabetes Journals From 2011 to 2013 Following CONSORT Statement: A System Review

**DOI:** 10.1097/MD.0000000000001083

**Published:** 2015-07-13

**Authors:** Xiao Zhai, Yiran Wang, Qingchun Mu, Xiao Chen, Qin Huang, Qijin Wang, Ming Li

**Affiliations:** From Graduate Management Unit, Changhai Hospital Affiliated to the Second Military Medical University, Shanghai, PR China (XZ and YW); Department of Orthopedics, Changhai Hospital Affiliated to the Second Military Medical University, Shanghai, PR China (XZ and ML); Department of Endocrinology, Changhai Hospital Affiliated to the Second Military Medical University, Shanghai, PR China (QH and QW); Department of Neurosurgery, the First Hospital of Jilin University, Jilin, PR China (QM); and Department of Neurosurgery, Hongqi Hospital of Mudanjiang Medical University, Heilongjiang, PR China (QM).

## Abstract

To appraise the current reporting methodological quality of randomized clinical trials (RCTs) in 3 leading diabetes journals.

We systematically searched the literature for RCTs in *Diabetes Care*, *Diabetes* and *Diabetologia* from 2011 to 2013.

Characteristics were extracted based on Consolidated Standards of Reporting Trials (CONSORT) statement. Generation of allocation, concealment of allocation, intention-to-treat (ITT) analysis and handling of dropouts were defined as primary outcome and “low risk of bias.” Sample size calculation, type of intervention, country, number of patients, funding source were also revealed and descriptively reported. Trials were compared among journals, study years, and other characters.

A total of 305 RCTs were enrolled in this study. One hundred eight (35.4%) trials reported adequate generation of allocation, 87 (28.5%) trials reported adequate concealment of allocation, 53 (23.8%) trials used ITT analysis, and 130 (58.3%) trials were adequate in handling of dropouts. Only 15 (4.9%) were “low risk of bias” trials. Studies at a large scale (n > 100) or from European presented with more “low risk of bias” trials than those at a small scale (n ≤ 100) or from other regions. No improvements were found in these 3 years.

This study shows that methodological reporting quality of RCTs in the major diabetes journals remains suboptimal. It can be further improved to meet and keep up with the standards of the CONSORT statement.

## Abstract 2

Supplemental digital content is available in the text

## INTRODUCTION

Randomized controlled trials (or randomized control trials, RCTs) are often used to determine the efficacy or effectiveness of different types of medical intervention. It is a gold standard for a clinical trial where the people being studied are randomly allocated.^1^ According to the US Preventive Services Task Force,^2^ at least 1 properly designed RCT should be included in the Level I evidence of the evidence-based medicine (EBM). Therefore, the quality of an RCT might influence the clinical decision made on EBM.

Poor quality of key methodological features in RCTs might lead to a misleading of inferior or harmful treatments.^3^ It is reported that trials without double-blinding may exaggerate intervention benefits by 14%,^4^ and flaws in the randomization can overestimate them by 30%.^5^ In addition, when meta reviews and guidelines include them without careful assessment, conclusions will be rigorously compromised.^6,7^ In this regard, a regular evaluation and check of methodological quality of clinical trials is essential.

To prevent such incidents, the Consolidated Standards of Reporting Trials (CONSORT) Statement was set as a standard, and was recently revised in 2010.^8^ The CONSORT was published in 1993 by 30 experts, which aimed to alleviate the problems arising from inadequate reporting of RCTs.^9^ Now it consists of 25-item checklist and a participant flow diagram, focusing on reporting how to design, analyze and interpret, and over 600 journals and editorial groups worldwide endorse it, including the *Lancet*, *BMJ*, *JAMA*, and *New England Journal of Medicine*. Authors should prepare reports of RCT in a complete and transparent way, reducing the influence of bias. Recent systematic reviews suggested that use of the CONSORT checklist was associated with improved reporting of RCTs.^10,11^

Under the guidance of CONSORT statement, assessment of the reported methodology of RCTs in various fields has been published previously, including surgery,^6,12^ gastroenterology,^13^ and nursing.^14^ However, evaluation of the methodological reporting quality of RCTs on diabetes has never been reported before.

Diabetes is a group of metabolic diseases in which blood sugar levels increased over a prolonged period.^15^ And in 2013, 382 million people suffered from diabetes worldwide.^16^ Researches on drugs and surgeries have been developed for a long time, and a growing number of RCTs have been put into practice, which become heavily weighed for screening effective agents and approaches.

To address this issue, we systematically appraised the methodological reporting quality of RCTs in 3 main diabetes journals from 2011 to 2013 following the CONSORT statement. We aimed to identify current strengths and weaknesses of methodological reporting quality of RCTs on diabetes.

## METHODS

The present study included all RCTs published as full text articles in *Diabetes Care*, *Diabetes* and *Diabetologia* from 2011 to 2013. We decided to study these 3 journals since they are leading diabetes journals focusing on diabetic diseases and their reported methodology has never been systematically studied. The ethical approval was not necessary since it was a literature review.

The methods used in this article were referred to those described previously.^6,13,17^ Shortly, trials were thought to be RCTs if the words “random,” “randomly,” or “randomization” were found in the text to describe the allocation method. In addition, trials published as abstracts, quasi-randomized trials, trials being part of some large RCTs, trials with animals or subgroups analysis of RCTs and observational studies nested within RCTs were excluded from the study.

Two cofirst authors hand-searched all the issues of the 3 journals published in 2011, 2012, and 2013 in the Pubmed database with the strategy by Robinson and Dickersin^18^ to include all potentially eligible trials. Relevant trials were then identified and analyzed.

Geographical, publishing, clinical, and epidemiological characteristics were extracted. Specifically, all studies were assessed according to a 9-item list that was adopted from Cochrane guidelines for methodological assessment^19^ and previous lists described by Ahmed Ali et al.^12^ These items are defined as follows:

1. *Primary outcome*: adequate if primary outcome stated explicitly.
2. *Sample size calculation*: adequate if presented.
3. *Presence of baseline*: adequate if basic information of patients in each study was described (at least 2 characteristics).
4. *Generation of allocation sequence*: adequate if the method was defined and was considered random beyond any doubt (eg, computer-generated sequence, random table, coin toss, or shuffle cards).
5. *Concealment of allocation*: adequate if a proper method to avoid knowing or expecting the allocation sequence in advance was confirmed to have been used (eg, central/pharmacy randomization, envelopes, or independent person).
6. *Blinding*: adequate if stating the use of any type of blinding of participants, outcome reviewers, researchers, or caregivers.
7. *Double-blinding*: adequate if stating that the study was double-blind.
8. *Type of analysis*: Intention-to-treat (ITT) if randomized patients with available data were accounted clearly as having been analyzed in their assignment group. Per-protocol analysis if only data of patients who have finished the routine were reported.
9. *Handling of dropouts*: adequate if <20% of trial participants were lost to follow-up, and reasons for all losses to follow-up were stated.

Criteria for “low risk of bias” trials were similar to reported previously, and were based on the available empirical evidence signifying their direct influence on effect estimates.^7,12,20^ The 4 criteria were defined as following: adequate generation of allocation, adequate concealment of allocation, ITT analysis, and adequate handling of dropouts.

The agreement of the 2 authors (X.Z. and Y.R.W.) was rated by calculation of kappa value. Any disagreement was resolved by discussion between the 2 reviewers. If the discrepancies could not be resolved by conversation, then the opinion of senior reviewer (Q.J.W. or M.L.) was sought. The primary aim of this study was to illustrate the current quality of the methodology reported in 3 major diabetes journals.

We performed an 1-way ANOVA followed by SNK test for strata comparisons. Descriptive statistics (mean, standard deviation) were used. All statistical analyses were performed with SAS 9.1 software. A *P* < 0.05 was regarded statistically significant.

## RESULTS

A total of 404 studies were retrieved from the 3 journals. Of these 404 studies, 99 were excluded because they had a nonrandomized design, subgroup analyses, letters or reviews, a pooled analysis of RCTs or cost-effective studies alongside RCTs. Finally, 305 trials were suitable for the analysis including 38 from the Diabetes, 222 from the Diabetes Care, and 45 from Diabetologia. Ninety-eight trials were published in 2011, 81 were published in 2012 and 126 published in 2013. The details of the data were listed in the “Supplemental Digital Content,” http://links.lww.com/MD/A317.

Major characteristics of the included trials are shown in Table 1. Generally, the 305 trials included a median of 103 patients (25th percentile 31, 75th percentile 328). Two hundred fifty-four (83.3%) trials published specified primary positive outcomes and 51 (16.6%) trials reported results of no differences. In addition, none of negative results were reported. Nearly half of all the trials were reported from Europe (44.6%, 136/305), with the United States reporting 39.3% (120/305), and Asia contributing (11.8%, 36/305) trials.

**TABLE 1:**
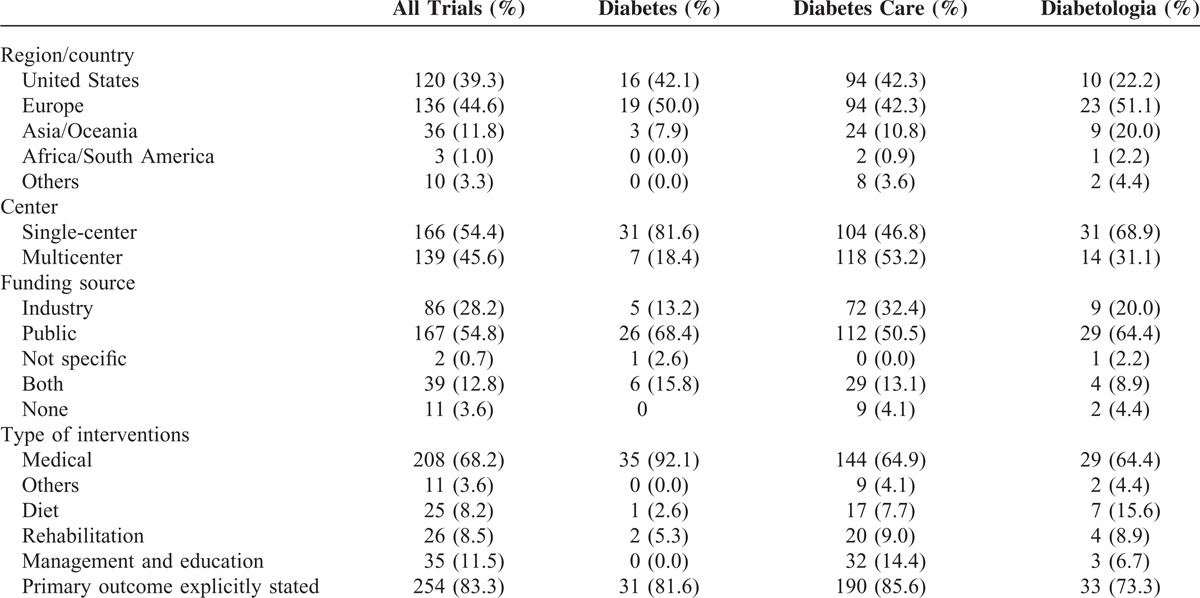
Principal Characteristics of the Included Trials

Kappa values for the interobserver agreement between the 2 reviewers were calculated: 0.91 for the generation of the allocation sequence, 0.90 for the allocation concealment, 0.87 for the ITT analysis, and 0.89 for handling of dropouts, 0.91 for the double blinding and 0.92 for the sample size calculation. All these values indicated almost perfect or substantial agreement.

For methodological reporting quality, the generation of the allocation sequence was adequate in less than half trials (108, 35.4%). The allocation concealment was adequate in 28.5% trials. Adequate blinding was reported in 57.0% trials including 129 (42.3%) double-blinding stated trials. However, 131 (43.0%) trials report inadequate blinding. Two hundred twenty-three (73.1%) trials reported dropouts, but only 130 (58.3%) trials were adequate in handing of dropouts and 53 (23.8%) trials used ITT analysis. Very few studies (15, 4.9%) were defined as “low risk of bias” trials (Table 2).

**TABLE 2:**
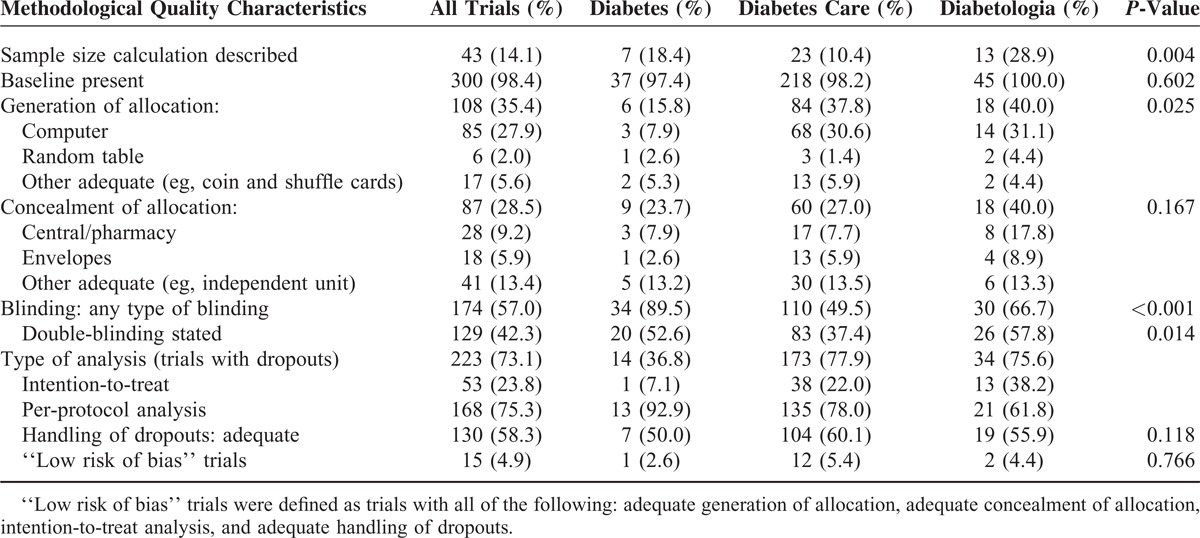
Methodological Reporting Quality of RCTs in Three Major Diabetes Journals From 2011 to 2013

According to different strata, it was found generally that large scale (n > 100) and European studies had more “low risk of bias” trials than small scale (n ≤ 100) and other regional studies. No improvements were shown in these 3 years. On the other hand, single-center studies had better quality of reported methodology for RCTs in allocation sequence generation, while multicenter studies were better in ITT analysis. The quality of the reported methodology is summarized in Table 3.

**TABLE 3:**
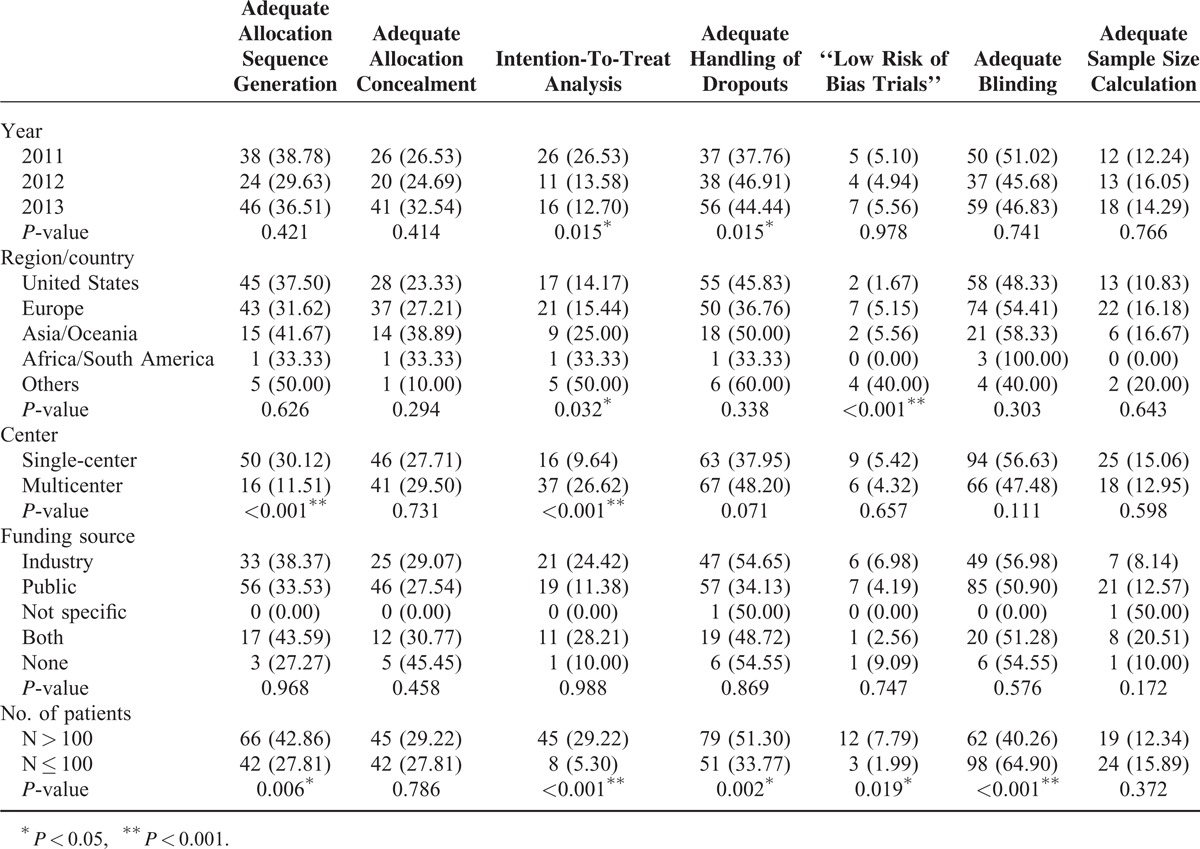
Methodological Reporting Quality of RCTs in 3 Major Diabetes Journals From 2011 to 2013 According to Different Strata

## DISCUSSION

In the present study, we described the current methodological reporting quality in 3 major diabetes journals. It was noted that 64.6% of all RCTs did not report adequate generation of the allocation sequence, 71.5% did not report adequate allocation concealment, 76.2% did not perform ITT analysis, and 73.1% did not report adequate handling of dropouts. These findings suggest that more efforts can be given to improve the quality of the reported methodology for RCTs in major diabetes journals.

Evidence-based medicine has drifted in recent years, and an increasing number of RCTs has been carried out. In addition, risk assessment using “evidence based” scores and algorithms has been extended to an industrial scale.^21^ Since the conclusions of these studies are often accepted by future guidelines and could be severely compromised,^22,23^ it is vital to appraise the quality of methodology of these RCTs critically. However, reporting qualities of RCTs were not satisfying.^11^ Therefore, this raised an imperative for publishing standards. Journal editors may raise the bar for authors to improve the usability of evidence, and also require research findings to be presented in a way that notifies individualized conservations.^24^

In this study, we used the CONSORT Statement as a criterion. For the 3 diabetics journals and in their parts of the instruction of submission, *Diabetes Care* and *Diabetologia* require authors to submit a completed CONSORT checklist with the manuscript while *Diabetes* does not explicitly claim for it.

Randomization-based inference is a fundamental principle in experimental design and in survey sampling. Randomization reduces confounding by equalizing independent variables that have not been accounted for in the experimental design. According to Lachin,^25^ an ideal randomization procedure would set the goals for achieving maximization of statistical power, minimization of selection bias, and minimization of allocation bias. In addition, the process of randomization initiates with this sequence generation process. In this study, a trial considered with adequate sequence generation should clearly report the exact methods, such as using a computer random number generator, a random number table, or other processes. Sequence generation based on such simple statements as “using a randomized design” or “we randomly allocated,” was regarded inadequate. As a result, 35.4% RCTs reported adequate sequence generation, including 27.9% by computer-generated sequences, 2% by random tables and 5.6% by coins or shuffle cards. Significant differences were shown among the 3 diabetes journals, and *Diabetes Care* and *Diabetologia* reported better methodology than *Diabetes*. However, nearly 64.6% of total RCTs did not report adequate sequence generation, signifying possibly inadequate randomization process. It may cause misleading and introduce significant biases and should be avoided in the future study. Further, trials performed by single centers (*P* < 0.001) and with a large number of patients (*P* = 0.006) did better in sequence generation. It seemed easier for a single center to perform adequate sequence generation.

In practice, it is difficult to maintain impartiality to take care of individual patients. Allocation concealment is important in RCTs because it protects the randomization and defeats patients and investigators from discovering treatment allocation until the study has concluded.^26^ It is therefore recommended that the allocation concealment methods should be reported in detail not only in an RCT's protocol but also in the publication of its results.^27^ Standard methods include central/pharmacy controlled randomization; sequentially numbered, opaque, sealed envelopes; and an independent unit.^28^ Our study found that 28.5% of trials reported adequate allocation concealment. It was good to see the constituent ratio of central/pharmacy controlled randomization (9.2%) and an independent unit (13.4%) surpassed that of sealed envelopes (5.9%) since it is suggested that the envelope method be more vulnerable to manipulation than other approaches.^29^ However, 71.5% of trials in this study were reported with inadequate allocation concealment. Wood et al^20^ conducted a meta-epidemiological study and concluded that the results of RCTs with inadequate or unclear allocation concealment seemed to be biased. As a result, editors and authors should pay more attention to allocation concealment.

An ITT analysis of results is based on the initial treatment assignment and not the eventual treatment consequence. It is intended to avoid various misleading effects of crossover and dropout, which may break the random assignment to treatment groups in a study. Since it started in the 1960s, the principle of ITT has become widely accepted for the analysis of controlled clinical trials. In contrast, a per-protocol analysis involves only patients who complete the entire clinical trial according to the protocol, and it may over evaluate the practical value of the new drug. As a result, all other analyses than ITT can introduce bias due to an imbalance of these factors. In this study, only 23.8% trials were reported using ITT analysis, which was far away from the standard of the CONSORT. Further, we found that larger multicenter trials might be more reliable than smaller a single-center trials. In addition, the number of trials using ITT published in 2011 was the biggest among the 3 years, and it reduced in 2012 and 2013. The reason may be that both authors and editors raised attentions to the CONSORT that carried out in 2010, but they gradually slacked as time passed by. As a result, they should notify the insufficient attention.

Missing data caused by patients dropping out of the study before completion is a major problem in the analysis of clinical trials. It can result in biased treatment comparison and influence the overall statistical power of the study.^30^ So, it is important to report an adequate handling of dropouts since it could notify readers unsatisfactory reasons for dropouts including adverse events, lack of efficacy, lost to follow-up, death, and so on. In our study, 58.3% of trials reported an adequate handling of dropouts, and there was a significant improvement since 2011 (*P* = 0.015). Trials with a large scale seem to be significantly better in this field (*P* = 0.002).

A blind or blinded study is an experiment in which the tester, the subject, or both, is unaware of information about the test that might lead to bias in the results. If both investigators and subjects are blinded, the trial is a double-blind experiment. In this study, only 57% of trials used adequate blinding and 42.3% of trials used double-blinding. There is significant difference among 3 journals, and *Diabetes* did the best for reporting adequate blinding while *Diabetologia* published the most trials stating double-blinding.

Adequate sample size calculation demonstrates how well the trial is designed. When sample size is smaller than needed, it is easy to draw negative conclusions and when it is larger, it will cost extra time and money. For the 305 trials in this study, the median sample size was 103 patients which was relatively small. And only 14.1% of the trials clearly specified the process of sample size calculation. *Diabetologia* did significantly the best among the 3 journals (*P* = 0.004). It can be improved further.

The “low risk of bias” trials were defined by 4 criteria including adequate generation of allocation, adequate concealment of allocation, ITT analysis and adequate handling of dropouts. Only 15 trials (4.9%) are in line with the criteria. The highest proportion of low risk of bias trials was from Europe (7 trials), whereas the lowest was from United States. Trials at a large scale showed more low risk of bias trials (*P* = 0.019). It indicates that the methodological quality of RCTs published in journals of diabetes field can be improved to reduce high risks of bias.

In other medical disciplines, methodological quality of RCTs was assessed with the similar method. Ahmed Ali et al^12^ compared the reporting quality of RCTs in surgical RCTs published in 1999 and 2009. They included 750 trials and found that methodological quality of surgical trials in 2009 improved in terms of sample size calculation (from 34% to 48%), adequate generation of randomization sequence (from 32% to 47%), concealment of randomization sequence (from 32% to 50%), and use of ITT analysis (from 20% to 33%) as compared with 1999 (*P* < 0.001 for all). The proportion of low risk of bias trials increased from 6% to 14% (prevalence ratio 2.59; 95% confidence interval 1.55–4.32). Bai et al^17^ reported methodological quality of RCTs of gastrointestinal and surgical endoscopy journals. Fifty percent (32/64) of all trials reported adequate generation of the allocation sequence, 58% (37/64) reported adequate allocation concealment, 47% (30/64) reported adequate blinding, 47% (30/64) reported adequate sample size calculation, and 67% (43/64) failed to disclose the funding source. Comparatively speaking, RCTs quality in diabetes journals is not satisfactory. This is probably due to not enough attention paid to this field.

The present study had several limitations. First, only trials in recent 3 years were included. The study does not show the trend of the quality of these journals. We therefore provided the proportions. Second, this study focused on the reporting quality of methodological details, which may differ from the quality of actual study.^31^ That means a well-designed and well-conducted trial may be considered with high risk of bias if the methodological methods were inadequately reported. Third, to evaluate the quality of reporting in RCTs quantitatively, we extracted major item instead of all items from the CONSORT 2010 statements.^32^ Forth, although the 3 highest impact factor diabetes journals were considered, a number of diabetes related RCTs are published in other nondiabetic or lower impact journals. To some extent, this study reflected the reporting quality of RCTs in a better level of diabetes trials.

In summary, the present study showed that the quality of the methods of RCTs in 3 major diabetes journals can be further improved. We hope more attention could be paid by authors, journal editors, and readers to the reporting methodology of randomized trials to keep RCT as one of the best methods for achieving credible evidence in the field of diabetes diseases.
